# *In silico* vs. Over the Clouds: On-the-Fly Mental State Estimation of Aircraft Pilots, Using a Functional Near Infrared Spectroscopy Based Passive-BCI

**DOI:** 10.3389/fnhum.2018.00187

**Published:** 2018-05-17

**Authors:** Thibault Gateau, Hasan Ayaz, Frédéric Dehais

**Affiliations:** ^1^ISAE-SUPAERO, Institut Supérieur de l'Aéronautique et de l'Espace, Université Fédérale de Midi-Pyrénées, Toulouse, France; ^2^School of Biomedical Engineering, Science Health Systems, Drexel University, Philadelphia, PA, United States

**Keywords:** fNIRS, BCI, working memory, prefrontal cortex, simulated and real flight, neuroergonomics

## Abstract

There is growing interest for implementing tools to monitor cognitive performance in naturalistic work and everyday life settings. The emerging field of research, known as neuroergonomics, promotes the use of wearable and portable brain monitoring sensors such as functional near infrared spectroscopy (fNIRS) to investigate cortical activity in a variety of human tasks out of the laboratory. The objective of this study was to implement an on-line passive fNIRS-based brain computer interface to discriminate two levels of working memory load during highly ecological aircraft piloting tasks. Twenty eight recruited pilots were equally split into two groups (flight simulator vs. real aircraft). In both cases, identical approaches and experimental stimuli were used (serial memorization task, consisting in repeating series of pre-recorded air traffic control instructions, easy vs. hard). The results show pilots in the real flight condition committed more errors and had higher anterior prefrontal cortex activation than pilots in the simulator, when completing cognitively demanding tasks. Nevertheless, evaluation of single trial working memory load classification showed high accuracy (>76%) across both experimental conditions. The contributions here are two-fold. First, we demonstrate the feasibility of passively monitoring cognitive load in a realistic and complex situation (live piloting of an aircraft). In addition, the differences in performance and brain activity between the two experimental conditions underscore the need for ecologically-valid investigations.

## 1. Introduction

Neuroergonomics is an emerging field of interdisciplinary research that promotes the understanding of the brain in complex real-life activities. This approach merges knowledge and methods from cognitive psychology, system engineering, and neuroscience (Parasuraman and Wilson, 2008). Accurate and reliable mental state assessment of human operators during use of complex systems is a prime goal of neuroergonomics that aims to measure the “brain at work” (Parasuraman and Rizzo, 2008). Understanding the underlying neurocognitive processes of such interaction could be used to improve safety and efficiency of the overall human-machine pairing. This could be achieved by (i) the augmentation of human performance and its translation to improved functioning “at work”, (ii) informing the design of the complex systems, or (iii) adapting the user interface and task parameters dynamically during use.

Aviation operations constitute an ideal paradigm to implement this approach. Pilots deal with an uncertain environment and face complex interaction with the flightdeck (Causse et al., 2013; Çakır et al., 2016; Reynal et al., 2016). For instance, several studies have emphasized that pilots' working memory (WM) abilities are heavily recruited to handle flightpath, to monitor the flight parameters, and to maintain an up-to-date situation awareness (Causse et al., 2011a,b). WM is also an important component when following air traffic control (ATC) instructions (Morrow et al., 1993). This activity indeed requires mentally storing flight parameters (e.g., heading, altitude, speed) to follow the adequate flight path. However, it is well-known that human working memory is fundamentally limited (Baddeley, 1992; Miller, 1994) and easily overwhelmed when task demand is excessive (Durantin et al., 2014a). Human factor studies emphasized that a variety of environmental stressors may negatively impact pilots' ability to execute ATC clearances (Billings and Cheaney, 1981; Taylor et al., 1994, 2005; Scerbo et al., 2003; Risser et al., 2006; Rome et al., 2012; Dehais et al., 2017). Thus, the implementation of monitoring technology in the cockpit to infer a state of cognitive limitation could represent a promising approach to enhance flight safety (Roy et al., 2017; Verdière et al., 2018).

Indeed, the development of brain computer interface (BCI) technology provides interesting prospects to continuously monitor and take advantage of the brain dynamics and the neural mechanisms underlying cognition. Among the three categories of BCIs (active, reactive, and passive) (Zander and Kothe, 2011; Vecchiato et al., 2016), the first two types are aimed at transforming cerebral activity into messages or commands to voluntarily control distant apparatus (e.g., mouse cursor). Passive BCIs (pBCI) are of particular interest for neuroergonomic applications (Cutrell and Tan, 2008; Frey et al., 2017; Gramann et al., 2017). They allow the use of interpretation of unlabeled brain activity during a task to derive various mental states (Blankertz et al., 2010; Roy et al., 2013; Van Erp et al., 2015; Zander et al., 2017). These mental state-inference systems offer a unique insight into the development of the human-system interactions to overcome cognitive limitations (Zander and Kothe, 2011; Brouwer et al., 2013). While several pBCIs have been successfully implemented in driving (Dijksterhuis et al., 2013) and flight simulator (Gateau et al., 2015; Aricò et al., 2016; Çakır et al., 2016; Callan et al., 2016; Verdière et al., 2018), few have attempted to test these systems under more realistic settings. However, very few studies have attempted to test these adaptive systems under realistic settings (Callan et al., 2015).

Electroencephalography (EEG) is by far the most popular technique (George and Lécuyer, 2010; Borghini et al., 2017) in the BCI community as it has excellent qualities for monitoring cognitive states (Brouwer et al., 2012; Roy et al., 2013) including superior temporal resolution and has been used to monitor working memory (Roy et al., 2013; Mühl et al., 2014). However, the localization of sources from the EEG signal requires higher-density recordings and additional computation to solve the inverse problem that may not be amenable to critical operational situations such as flying real aircraft. In addition, setup time and susceptibility to motion artifacts should be considered for minimally intrusive deployment. Thus, the use of functional near infrared spectroscopy (fNIRS) has been gaining popularity recently as the sensors have been miniaturized, become portable and wireless (Ayaz et al., 2013; Strait et al., 2014; Naseer and Hong, 2015; Schudlo and Chau, 2015).

This brain activity monitoring technique uses near-infrared light absorption properties of hemoglobin to estimate local variations of cortical oxygenation changes (Villringer and Obrig, 2002; Ayaz et al., 2012). fNIRS has been successfully used to detect working memory solicitation (Li et al., 2005; Schreppel et al., 2008; Hirshfield et al., 2011; Gagnon et al., 2012; Herff et al., 2014; McKendrick et al., 2014; León-Doḿınguez et al., 2015; Unni et al., 2017). Despite its relatively low temporal resolution, fNIRS poses several advantages compared to more established traditional tools (Kikukawa et al., 2008; Piper et al., 2014; McKendrick et al., 2015; Davranche et al., 2016) such a relatively high spatial resolution (around 1 cm^2^ depending on the sensor geometry) and high signal-to-noise ratio as sensors are relatively more robust to motion artifacts (Huppert et al., 2009), eye-blinks and facial muscles (Izzetoglu et al., 2004). It is also possible to run experiments with active and mobile subjects and even outdoors (Piper et al., 2014; McKendrick et al., 2016). Specifically, it is less sensitive to noisy electromagnetic environment in the aircraft (radio transmission, radio-navigation beacons, GPS antenna, etc.) than EEG, making it a candidate to measure pilot's brain activity during real flight. As an emerging neuroimaging technique, we believe that it is important to investigate the capabilities of fNIRS and its utility in future applications.

The present study aims to develop an on-line fNIRS based pBCI for the assessment of working memory of aircraft pilot during real flight. Earlier studies demonstrate that fNIRS based measures BCI have been implemented. They rely on oxygenation changes in the prefrontal cortex (PFC) and can be used for measuring WM load (Schreppel et al., 2008; Ayaz et al., 2012; Gagnon et al., 2012; Durantin et al., 2014a,b). Here, a pilot-ATC interaction task, was designed with two contrasted levels of WM load. A Support Vector Machine (SVM) based classifier performing on-line for single trial WM load level discrimination was implemented. This classifier was first tested in a high fidelity flight simulator. The same machine learning approach was then utilized to assess the WM load level in an actual flight condition. To the authors' knowledge, this is the first study to monitor pilot's brain activity on-line under such operational settings and ecological validity. We also compared pilot's WM performance and related PFC activity both in high fidelity simulator and real flight conditions. The objective was to determine wether these two conditions simulated and real operational settings were equivalent or not in terms of task demand (Dahlstrom and Nahlinder, 2009; Batula et al., 2017). As most aviation psychology experiments and pilots' training are conducted with flight simulators, such assessment is critical for future design and development of such approaches (Philip et al., 2005).

## 2. Materials and methods

### 2.1. Passive BCI in flight simulator

#### 2.1.1. Participants

Fourteen visual flight rules (VFR) pilots (three women; mean group age: 29.25 ± 6.92; mean flight hours 80 ± 50) completed the experiment. Pilots had normal or corrected-to-normal vision, normal hearing, and no psychiatric disorders. They all had medical clearance to fly. After providing written informed consent, they were instructed to complete task training. The data from two participants were rejected due to a high level of fatigue in one case, and data collection issue for the second. Typical total duration of a subject's session (informed consent approval, practice task, and real task) was about two hours. This work was approved by the Institutional Review Board (IRB) of the Inserm Committee of Ethics Evaluation (CEEI: Comité d'Evaluation Ethique de l'Inserm IRB00003888). The methods were carried out in accordance with approved guidelines and participants gave written informed consent approved by the IRB of CEEI.

#### 2.1.2. Neurophysiological measurements: fNIRS

**Figure 1:**
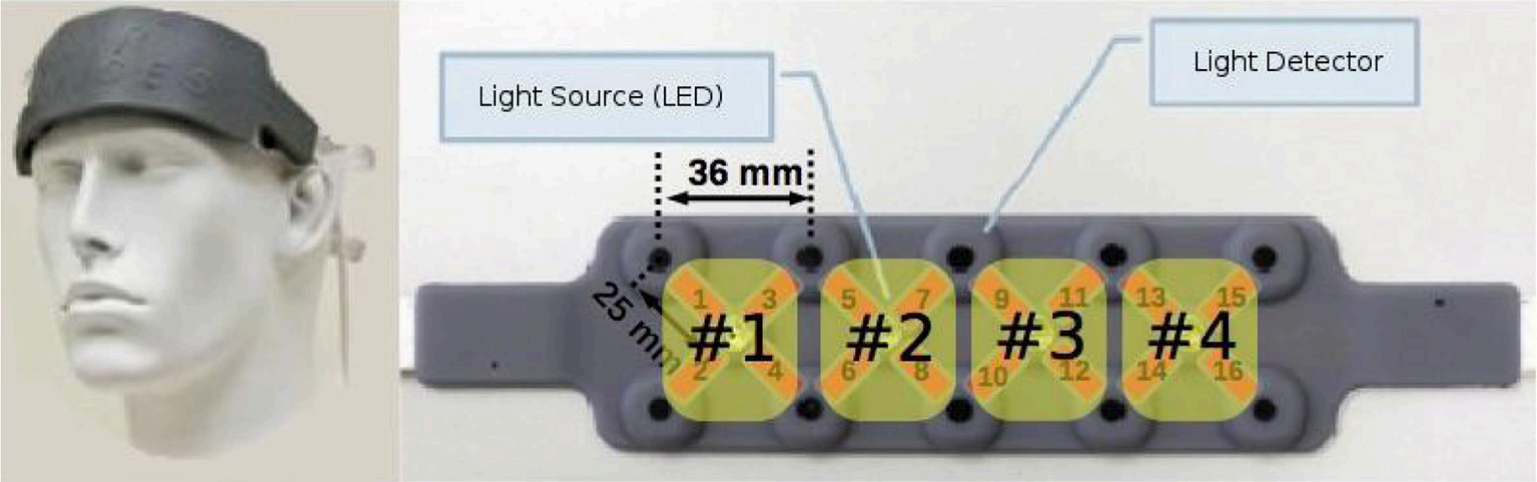
fNIR Device® Model 1200S headband and associated optode numbering. Only the four closest detectors to an emitter constituted optodes. Optodes are represented in red with their associated number. Four regions of interest (ROI) were defined for statistical analyses purposes (#1, #2, #3, #4).

During this experiment, we recorded hemodynamics of the prefrontal cortex using the functional near-infrared spectrometer fNIR Device Model 100B (Biopac®) equipped with 16 optodes (Figure 1). On this continuous-wave system, the optode separation was about 25 mm and two wavelengths were used, 730 and 850 nm. DPF (differential pathlength factor) value was 5.97 which is within the range used by many in literature (Kato et al., 1993; Luo et al., 2002) and accepted by many groups. Four regions of interest (ROI) were defined to allow for explorative statistical comparisons with the data collected during the real flight experiment (see section 3).

Each optode of the device records hemodynamics at a frequency of 2 Hz in terms of oxygenation level variations in comparison to an initial baseline performed prior to the experiment. Changes in the concentrations of oxygenated (Δ[*HbO*_2_]) and deoxygenated hemoglobin (Δ[*hHb*]) relative to the baseline can be calculated from changes in detected light intensity using the modified Beer-Lambert Law (Delpy et al., 1988). Cognitive Optical Brain Imaging (COBI) Studio® software (Ayaz and Onaral, 2005; Ayaz et al., 2011) was used to collect data. The data stream was available on-line from a TCP/IP interface. Before recording, signals for each optode were carefully checked for saturation with COBI Studio which provides signal quality visual representation. COBI studio was also used to check signal quality and to adjust consequently the headband on the participant's forehead. After this check, a baseline was established, which simply consists of letting the participant rest for 10 s.

#### 2.1.3. Experimental environment: flight simulator

**Figure 2:**
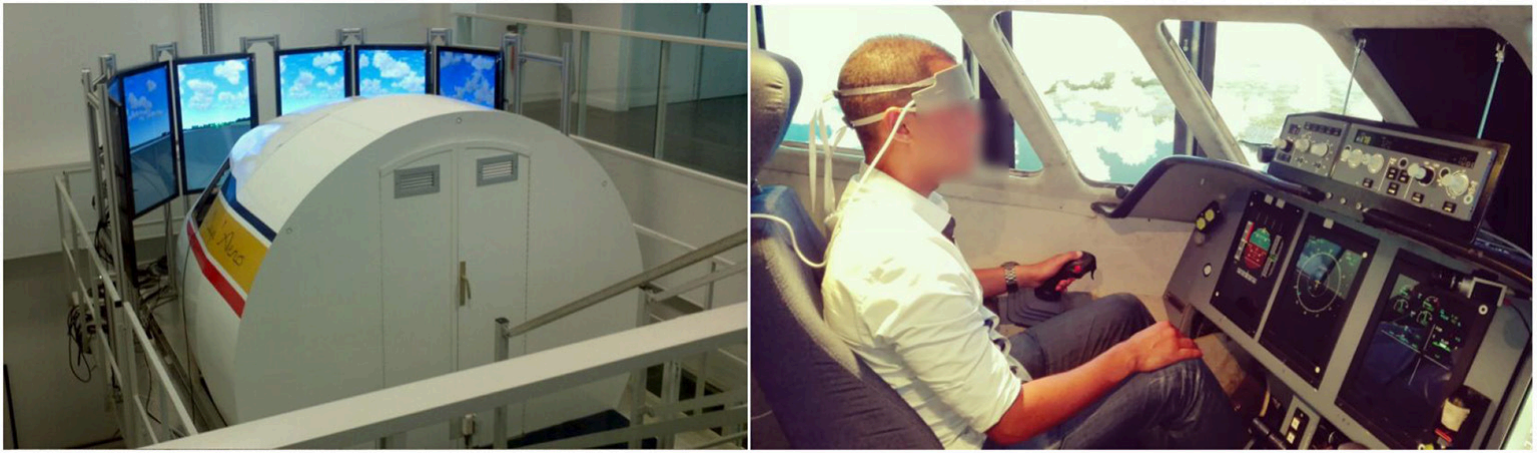
**(Left)** ISAE-Supaero flight simulator; the closed cabin is visible from rear, and eight screens are used to visualize external environment. **(Right)** The pilot subject with the fNIRS headband.

We used the ISAE-SUPAERO (Institut Supérieur de l'Aéronautique et de l'Espace - French Aeronautical University in Toulouse, France) flight simulator to conduct the experiment in an ecological situation. Its user interface is composed of a Primary Flight Display, a Navigation Display, and an Electronic Central Aircraft Monitoring Display (Figure 2).

#### 2.1.4. Task description

This protocol was adapted from a previous study (Gateau et al., 2015). As in real flight operations, pilots heard ATC instructions (pre-recorded for this experiment) to vector them and were asked to read back the instructions. Their answers were recorded for off-line behavioral analysis. The ATC messages were delivered at 78 dB through a Sennheiser® headset. Two levels of difficulty were defined based on the flight parameters that the participant had to read back during the experiment:

- Low WM load: The two first digits were the same for each flight parameter (e.g., 14 for “speed 140, heading 140, altitude 1400, vertical speed +1400”).
- High WM load: each flight parameter value was different from the previous one and composed of different number to increase task difficulty (e.g., “speed 172, heading 238, altitude 6400, vertical speed −2800”).

The task consisted of 10 repetitions of each difficulty for a total of 20 trials. The task difficulty order was randomly distributed with two constraints:

- the first 10 trials contained both 5 trials of high difficulty, and 5 trials of low difficulty (which is necessary for machine learning purposes, see section 2.1.5);
- the difficulty cannot be the same for more than two successive trials.

**Figure 3:**
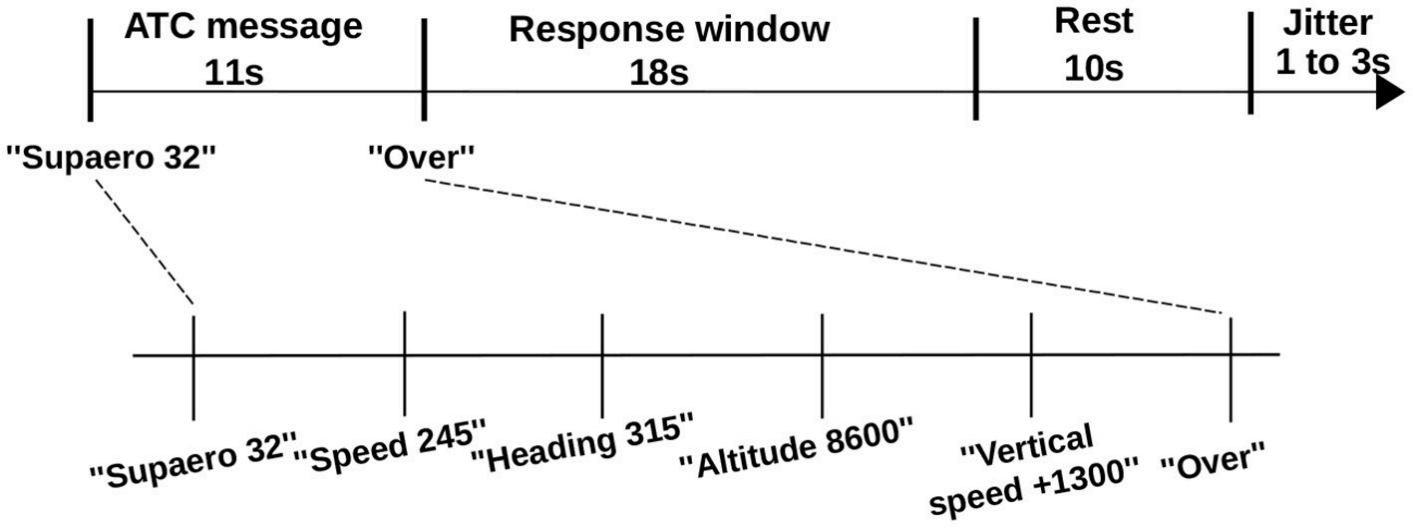
ATC span task trial design (High Load message).

Each ATC message started with the airplane call sign (i.e., “Supaero 32”), immediately followed by a sequence of flight parameters and ended with the message “over” (Figure 3). Thereafter, pilots had a 18 *s* response window to repeat the instruction. A practice session was conducted prior to the experiment runs to familiarize them with the experiment protocol and the interface.

During the experiment, the experimenter was collecting the volunteer's ability to read back each message so as to compute the total number of correct responses in the low and hard conditions.

#### 2.1.5. Experimental time course

**Figure 4:**
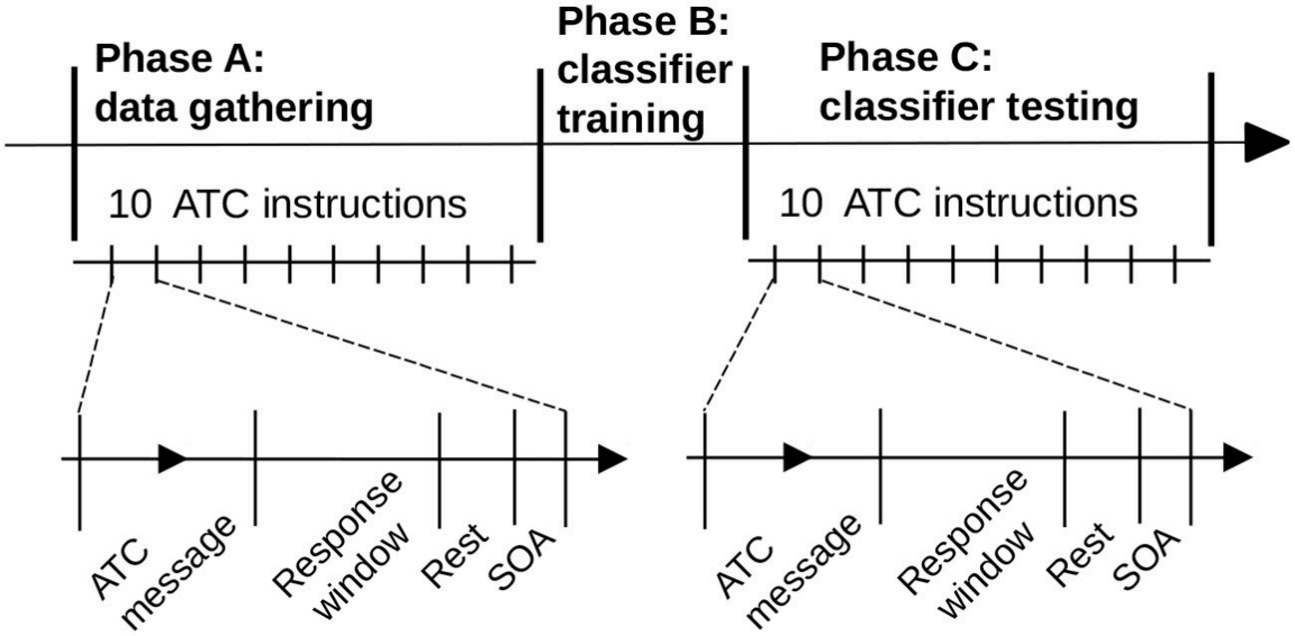
The experiment was split into three successive phases. Data gathering (*phase A*) and classifier testing (*phase C*) consisted of 10 ATC instructions each. The pilot's classifier was trained between these two phases (*phase B*). The time scale of the figure is illustrative.

For machine learning purposes, the experiment was split into three successive phases (Figure 4):

- Phase A – data gathering phase: 10 instructions with two levels of difficulty were successively presented to the pilot in a random order. During phase A, the correctness of the pilot's response was also checked for further pilot performance analysis. The fNIRS's data were processed and recorded for each trial's response window. The levels of difficulty of the message were also recorded.
- Phase B – classifier training phase: the classifier training process was activated, based on the data gathered during phase A. This phase was not perceived by the pilot and allowed further classification actions. At the end of this phase, the pilot's classifier - the pilot's specific classification model, correctly trained - was available for classification requests.
- Phase C – classifier testing phase: 10 instructions with random levels of difficulty (high WM load or low WM load) were successively presented. The aim of the classification process was to discriminate the difficulty of the trial. After each response window of trials, the classifier returned WM load estimation of the trial.

Note that the transition (phase B) from phase A to phase C was not perceptible to the participants.

#### 2.1.6. MACD filter

Raw fNIRS data were real-time filtered using a MACD filter, commonly used in economic market analysis (Appel, 2005). This filter, based on the difference between a short-term EMA (Exponential Moving Average) and a long-term EMA, implements a second order band-pass filtering to eliminate low-frequency (< 0.02 Hz) and high-frequency (>0.33 Hz) components from the raw fNIRS signal (Utsugi et al., 2007). This low order filter has a quasi linear phase in its bandwith and is particularly suited for real-time applications. For the experiment, we proceeded to an on-line filtering of Δ[*HbO*_2_] and Δ[*hHb*] on 16 optodes.

*N* represents the number of time points defining the EMA window:

(1) $$\begin{array}{l} y=EMA_{N}\left(x\right)\Leftrightarrow y_{n}=\frac{2}{N+1}x_{n}+\frac{N-1}{N+1}y_{n-1} \end{array}$$

(2) $$\begin{array}{l} MACD_{N_{short},N_{long}}\left(x\right)=EMA_{N_{short}}\left(x\right)-EMA_{N_{long}}\left(x\right) \end{array}$$

We chose a 6 s short-term EMA and a 13 s long-term EMA according to previous work (Durantin et al., 2014b) for MACD filtering, to get the desired bandwidth.

#### 2.1.7. Single trial SVM-based WM load estimation

The classification's goal was to discriminate on-line whether the last trial was a high WM load trial or a low WM load trial. For each pilot, we used the first 10 trials to train the pilot's classifier (phase A and B, see section 2.1.5). From trial 11 to 20, we used the pilot's classifier to discriminate trial difficulty, without any further training. An accuracy score (sum of correct predictions divided by total number of predictions) of the pilot's classifier was provided at the end of the experimental session.

**Figure 5:**
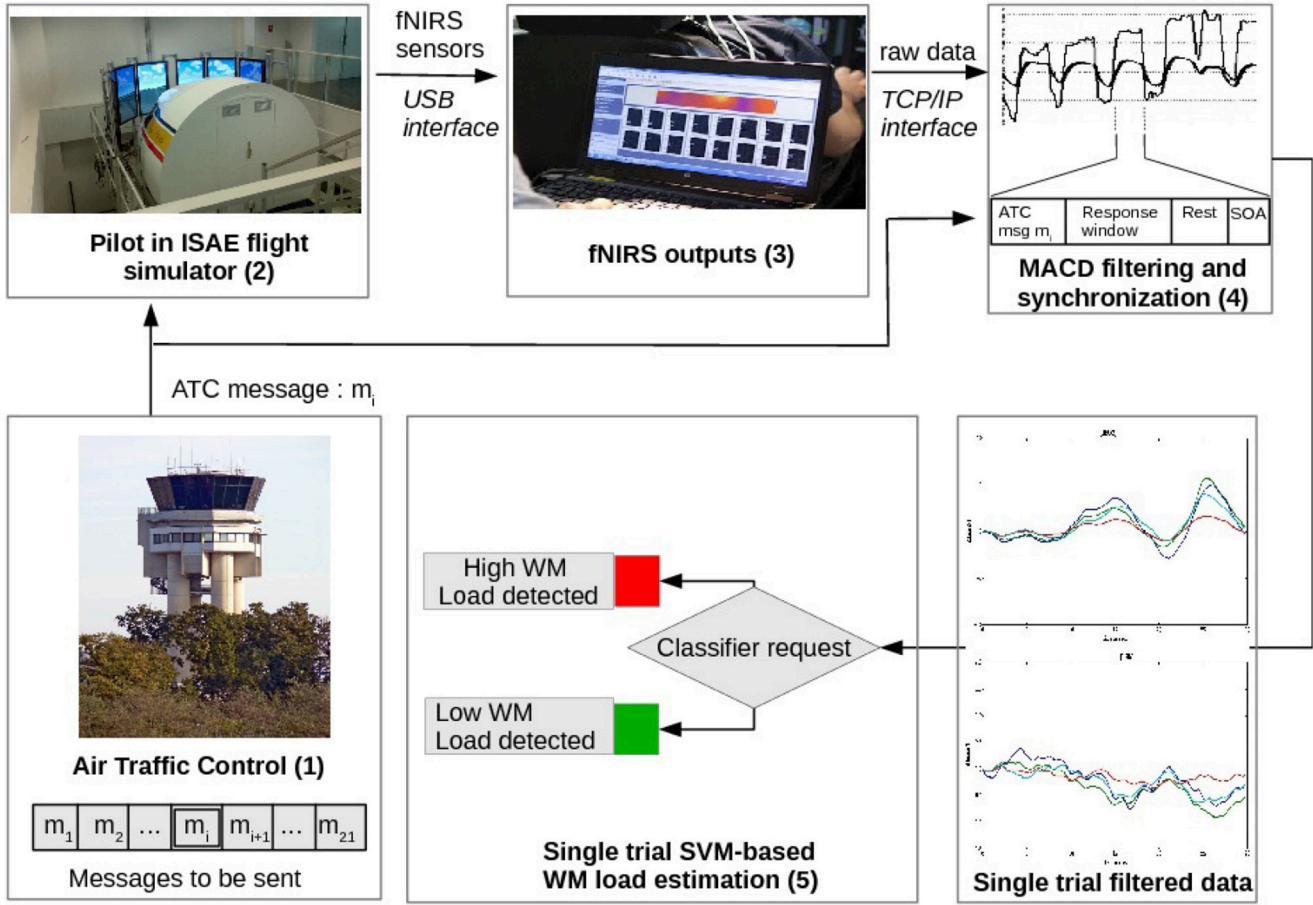
Illustration of the fNIRS based inference system. Pre-recorded ATC messages were sent to the pilot (1). The pilot's prefrontal activity was measured with a fNIRS device (2). Output measures (3) were MACD-filtered and synchronized with the temporal design of the trial (4). When all of the required data were available for the trial, a request was sent to the pilot's classifier to assess the WM load of the trial (5).

Sixteen optodes of Δ[*HbO*_2_] and Δ[*hHb*] filtered signals were segmented into trials, in real-time, according to the task synchronization module (Figure 5). Each trial starts when an ATC message is played, and lasts 30 s. All data points of a trial – 2 different inputs per optode, 16 optodes, 30 s of data with a 2 Hz sampling corresponding to 1920 features – were considered as the input of the machine learning process. A 30 s sliding window was chosen to be consistent with literature regarding inter-subject variability (Jasdzewski et al., 2003; Sato et al., 2005). Note that the transition from the “Response” phase to the “Rest” one was unnoticeable, as it was anticipated that participants started to rest as soon as they completed the instruction.

As our number of features was large compared to the training sample, we used a linear Support Vector Machine (SVM) (Cortes and Vapnik, 1995). The principle of the SVM is to find the separating hyperplane that maximizes the distance between the hyperplane and the closest training points in each class. To avoid over-fitting, we chose to customize the SVM regularization parameter for each pilot's classifier. In a linear SVM, the regularization parameter C controls the trade-off between errors of the SVM on training data and margin maximization. During the training process of each participant, the parameter C is incrementally changed over a large range of values (from 10^−3^ to 10^4^) with a 10-step factor. Hence, a five-fold cross-validation on the first 10 trials with scikit-learn (Pedregosa et al., 2011) packages (sklearn.svm and sklearn.cross_validation) was ran to select the C parameter with the highest performance in terms of accuracy. The classifier training (phase B) was performed as soon as the data of the first 10 trials were available for online purposes (Aricò et al., 2016).

#### 2.1.8. Experimental components' architecture

We implemented a WM load estimator that integrated different components (Figure 5):

- a simulated ATC which broadcasts a list of chosen messages to the pilot;
- the ISAE flight simulator (Figure 2);
- a fNIR sensor which measures the prefrontal oxygenation (Figure 1);
- a MACD filter for artifact removal (see section 2.1.6);
- a synchronization module that also formats filtered data for the classification process: filtered fNIRS output must be synchronized with the pilot's state, according to the instant of the arrival of that incoming message and according to the pilot's response window;
- a classifier (see section 2.1.7) which evaluates in real-time whether the last ATC instruction was a high WM load trial or a low WM load trial. Results were logged into a file, while a real time feedback is provided through a system terminal.

**Figure 6:**
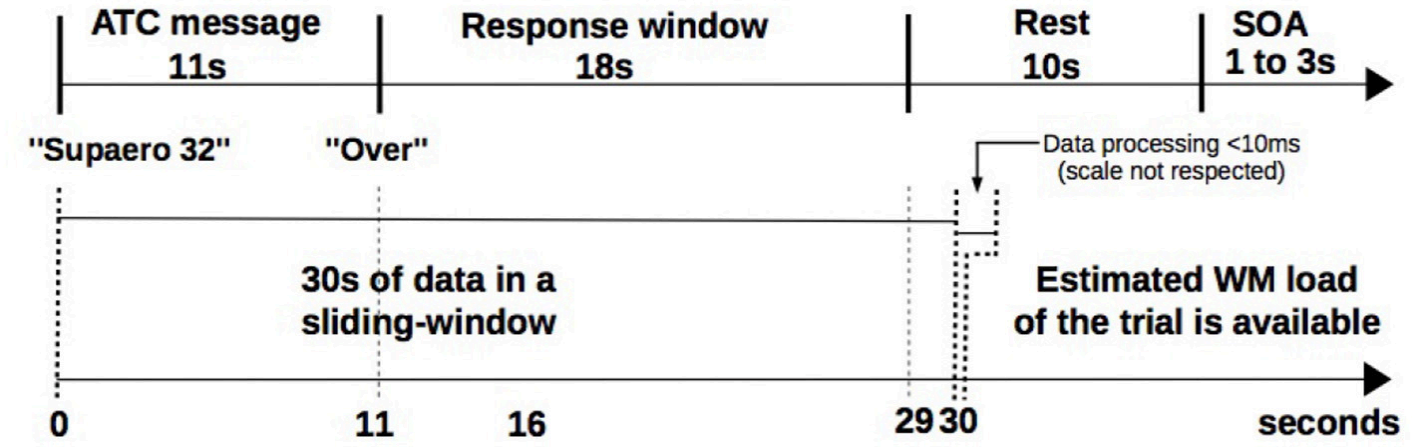
Trial timeline and computing latencies. Upper timeline shows ATC span task trial events duration (see Figure 3). Bottom timeline illustrates duration constraints to get pilot's estimated WM load: classifier's response is available in the worst case less than 10 ms after pilot's response window.

Task monitoring, data acquisition, and computation were conducted on the same computer (core i5-3210M, 2.50 GHz, 4 GB RAM). During the experiment, the classifier training (phase B) duration was short (800 ms) and remained unnoticeable for the participant. The classifier testing phase lasted 10 ms and was also unnoticeable for the participant” (Figure 6).

### 2.2. Passive BCI in real flight

#### 2.2.1. Participants

Fourteen VFR pilots (1 women; mean group age: 23.07 ± 5.35; mean flight hours 44.07 ± 37.52), completed the experiment. Please note that these volunteers were different from the ones who participated to the flight simulator experiment. The data from two participants were rejected due to light saturation issues and a device synchronization issue. After providing informed consent, they were instructed to complete task training on the ground. None of the recruited subjects had neurological or psychiatric history or was on medication. Each of them gave written informed consent for the experiment. The experimental protocol was approved by the committee of the European Aviation Safety Agency (EASA permit to fly approval number : 60049235). The methods were carried out in accordance with approved guidelines and participants gave written informed consent approved by the EASA.

#### 2.2.2. Neurophysiological measurements: mini-fNIRS

**Figure 7:**
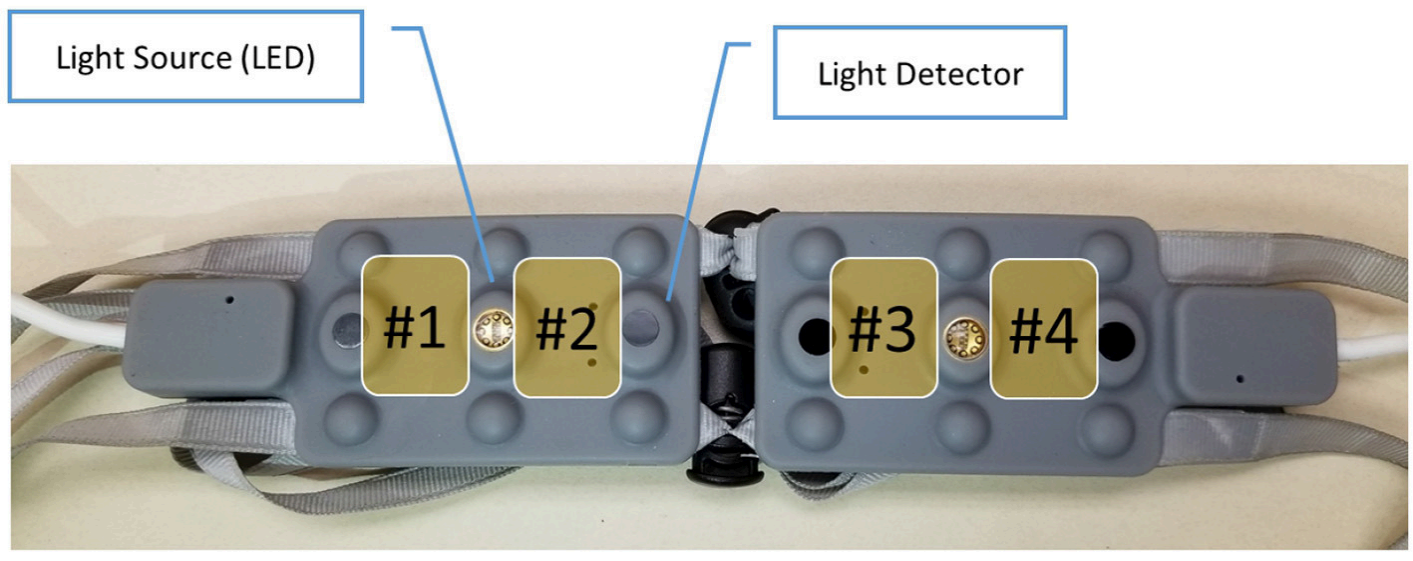
Miniaturized and wireless fNIR Device® Model 1200W headband and associated regions of interest (optodes). Optodes are represented in yellow with their associated number.

We used the miniaturized and wireless fNIR Device Model 1200W (Biopac®) portable system (Ayaz et al., 2013) to record the pilots' hemodynamics of the prefrontal cortex (Figure 7). This device was chosen as it was wireless (i.e., the pilot's head was not attached to any cables) and did not require external power supply as the Model 1200S. This was a prerequisite to facilitate its implementation and use in the aircraft for our experiment. This device had the same hardware design, and exactly same LED light source components and detectors than the fNIRS Model 1200S used in the flight simulator. Consistent with the previous device, on this continuous-wave system, the optode separation was about 25 mm and two wavelengths were used, 730 and 850 nm. The DPF value was 5.97. This four-optode device records hemodynamics at a frequency of 4 Hz in terms of oxygenation level variations in comparison to a baseline same as the 1200S desktop version. With flexible circuit board and separation-adjustable split pads, the sensors were positioned to aim monitoring brain areas similar to the ROIs extracted from 1200S sensor. Changes in the concentrations of oxygenated (Δ[*HbO*_2_]) and deoxygenated hemoglobin (Δ[*hHb*]) can be calculated from changes in detected light intensity using the modified Beer-Lambert Law (Delpy et al., 1988).

Cognitive Optical Brain Imaging (COBI) Studio® software (Ayaz and Onaral, 2005; Ayaz et al., 2011) was used to collect data. The data stream was available on-line from a TCP/IP interface. Before recording, signals for each optode was carefully checked for saturation with COBI Studio which provided a visual representation of signal quality. An aluminum foil attached to a dark ski band band and a cap were placed over the mini-fNIRS to shield against ambient sunlight infrared.

Data was MACD filtered and we used a similar on-line Experimental Components' Architecture with the exception that we used a real plane instead of the flight simulator.

#### 2.2.3. Experimental environment: DR400 aircraft

**Figure 8:**
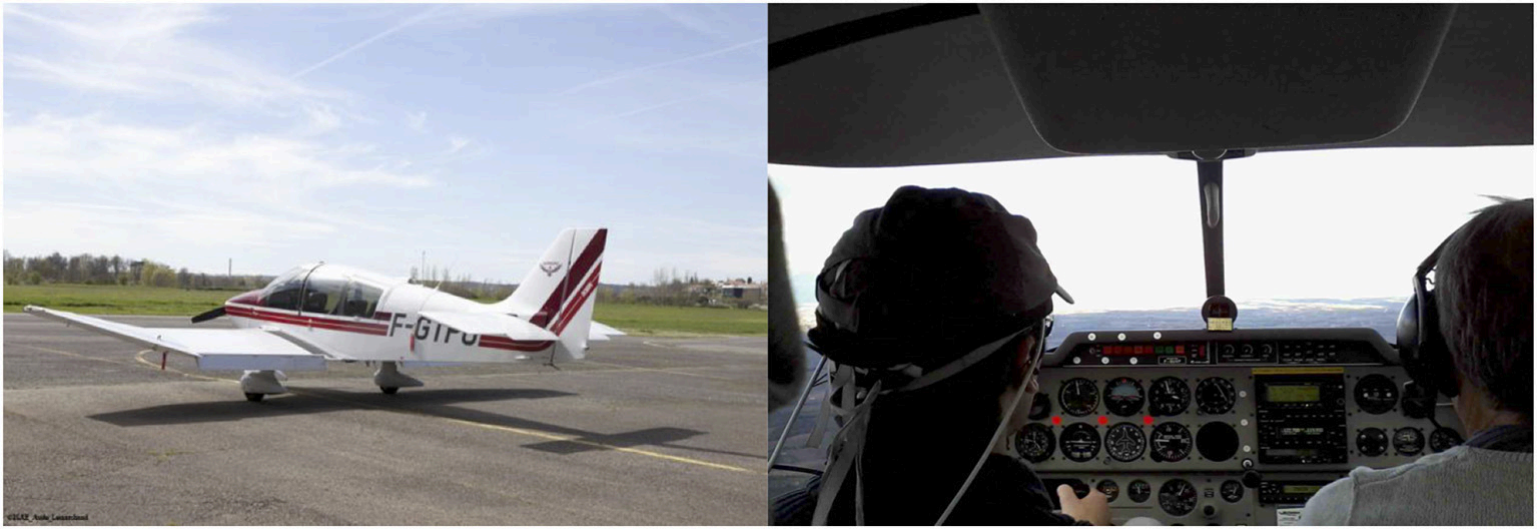
**(Left)** ISAE Supaero DR400 plane used for the experiment. **(Right)** The pilot subject with the mini-fNIRS headband (on the left) and the safety pilot (on the right).

The ISAE Supaero DR400 light aircraft was used for the purpose of the experiment (Figure 8). It was powered by a 180HP Lycoming engine and was equipped with classical gauges, radio and radio navigation equipment, and actuators such as rudder, stick, thrust, and switches to control the flight. The participant was placed on the left seat and was equipped with the mini fnirs system. The participant wore a Clarify Aloft® that was used to trigger task-related auditory stimuli from a PC via an audio cable. The participant could still communicate with the other crew members, real ATC and when he received auditory stimuli (emulated ATC). The safety pilot was an ISAE flight instructor. He was right seated and had the authority to stopping the experiment and taking over the control of the aircraft for any safety reason. The backseater was the experimenter: his role was to set the sensor, to trigger the experimental scenario and to supervise data collection.

#### 2.2.4. Task description

The experimental task and audio messages were similar to the previous protocol (see section 2.1), with the same experimental time course and the same instructions for the participant. A practice session on the ground was conducted prior to the experiment runs to familiarize them with the experiment protocol and the interface. After training was completed on the ground, the mini-fNIRS system was placed over the participants forehead. The participant then took off from Lasbordes (LFCL, Toulouse, France) airfield and began a local flight. The experimental task *per se* started when the pilot left the Lasbordes traffic pattern and was stabilized at an altitude of 2500 feet. The participant was asked to fly as straight and stable as possible and to only perform slight avoidance maneuvers as necessary. Once stabilized, the baseline of ten seconds was recorded. After the completion of the WM task, the participant was heading back to land at Lasbordes airfield. The total flight lasted one hour including the WM task.

As in simulated condition, the backseater was collecting the volunteer's ability to read back each message in order to compute the total number of correct responses in the low and hard conditions. These data allowed to compare the WM peformance accross the conditions (i.e., low vs. high; simulated vs. real flight).

#### 2.2.5. Experimental components' architecture and WM load estimation

We implemented a similar WM load estimator in the airplane as in the flight simulator. Machine learning inputs were lightly adjusted to fit the data flow available with the mini-fNIRS wireless portable device. The four (instead of 16) available optodes of Δ[*HbO*_2_] and Δ[*hHb*] filtered signals were segmented into trials, in real-time, according to the task synchronization module (see section 2.1.8). Each trial starts when an ATC message is played, and lasts 30 s. All data points of a trial - two different inputs per optode (i.e., Δ[*HbO*_2_] and Δ[*hHb*]), four optodes, 30 s of data with a 4 Hz sampling corresponding to 960 features - were considered as the input of the machine learning process.

### 2.3. Statistical analyses

Off-line statistical analyses were performed with “R” (R Core Team, 2013) software and the “EzANOVA” (Anderson, 2001) package to compare WM performance and prefrontal cortex activations in the flight simulator and in the real flight conditions during the 20 trials. Two-tailed unpaired t-tests were performed to compare the WM performance in the high and low load conditions across the two flight conditions (simulator and real flight). As the number of optodes was not equivalent between the two fNIRS devices (16 vs. 4), we defined four regions of interests (ROIs) for the fNIR100 device that was used in the simulator condition to allow for explorative comparisons with the real flight condition. ROI1, ROI2, ROI3, and ROI4 were derived respectively from the spatial averaging of optodes 1 to 4, 5 to 8, 9 to 12, and 13 to 16 (see Figure 1). The mean frontal Δ[*HbO*_2_] peak response and the mean frontal Δ[*hHb*] peak response (peak value within 30 s post-trial onset minus 2 s average pre-trial onset) over the four ROIs of the PFC for each trial and each pilot using the MACD-filtered data in both flight conditions (i.e., simulator and real flight) were computed. A multivariate analyses for repeated measures (MANOVA) was conducted over the mean Δ[*HbO*_2_] data with between factor flight condition (simulator vs. real flight) and within subject factors WM Load (High vs. Low) and ROI (#1, #2, #3 & #4); see Figure 1 was led. A similar MANOVA over the mean Δ[*hHb*] was then conducted. We then ran a two-tailed unpaired t-test to compare the classification accuracy in the two experimental conditions. The Tukey's Honestly Significant Difference (HSD) test was used for all *post-hoc* comparisons.

## 3. Results

### 3.1. Real flight vs. flight simulator: off-line behavioral and neurophysiological analyses

**Figure 9:**
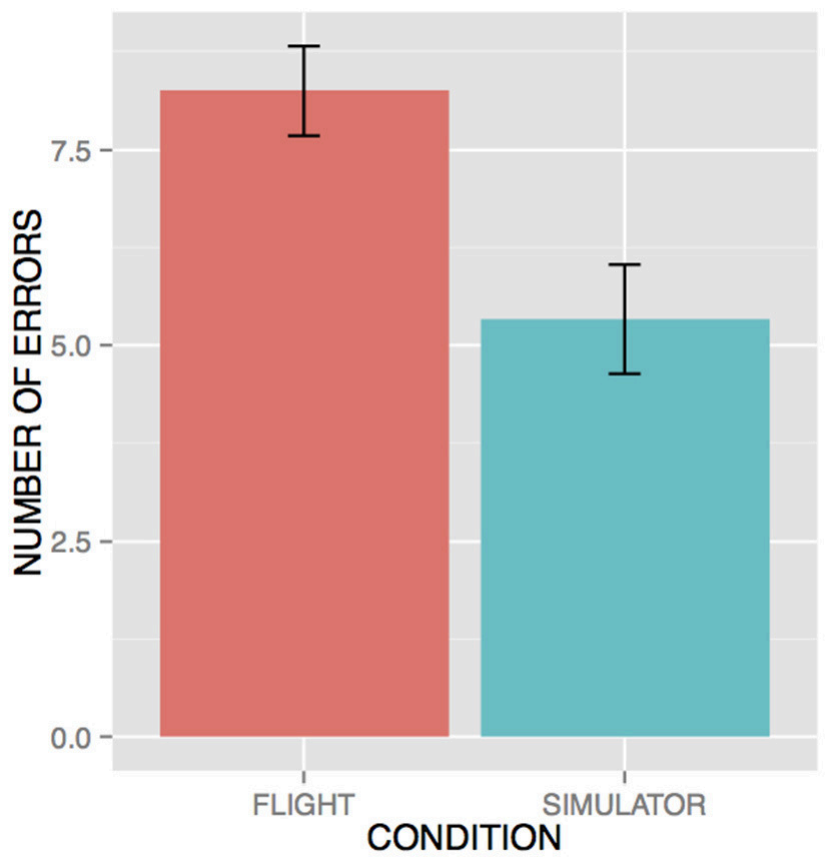
Behavioral performance in the high load WM condition. No errors occurred during the low load WM condition.

**Figure 10:**
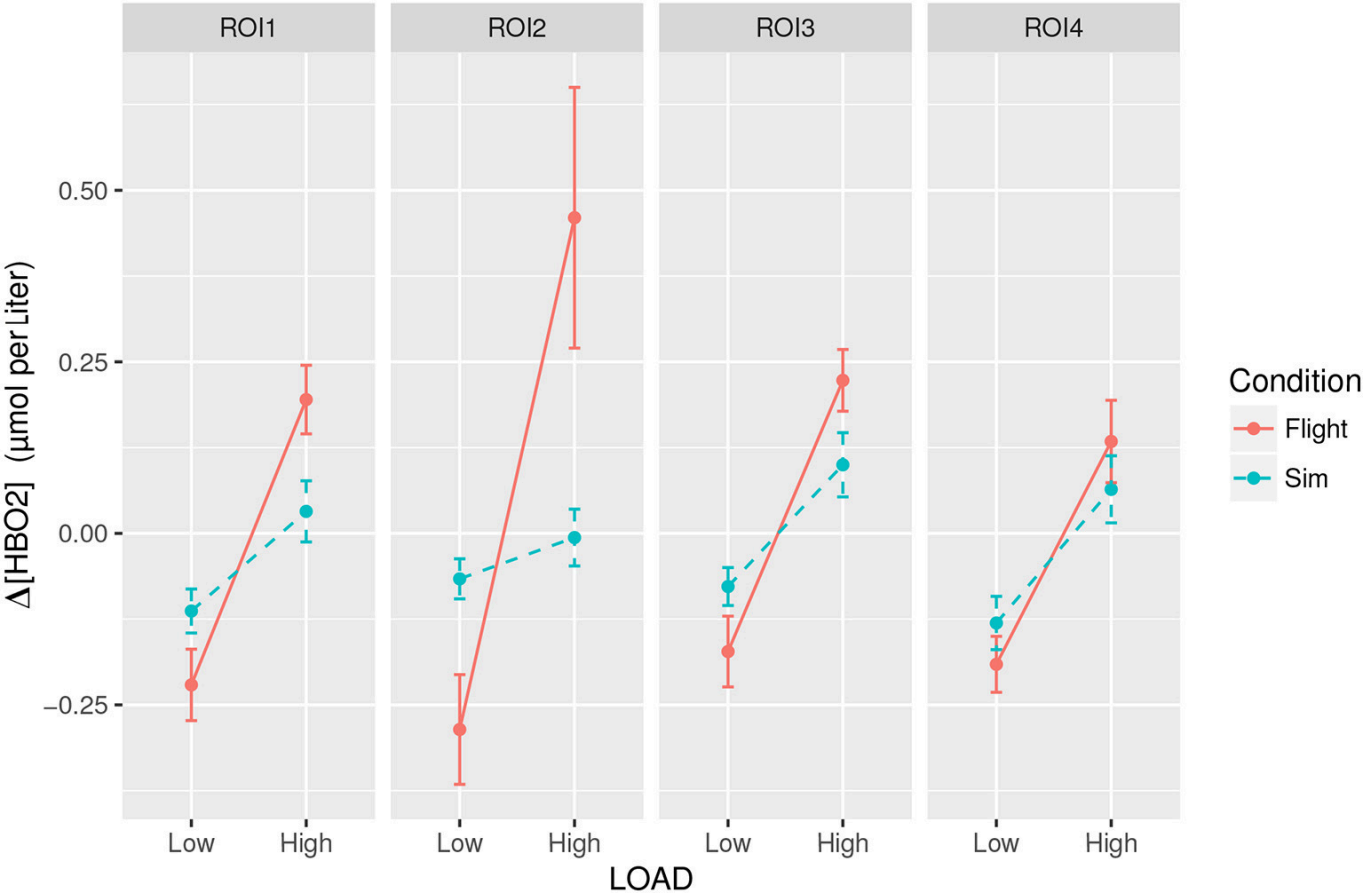
The mean frontal Δ[*HbO*_2_] peak response (peak value within 30 s post-trial onset minus 2 s average pre-trial onset) over the four ROIs of the PFC (from 1 - left to 4 - right) across WM loads and flight conditions.

Participants committed on average 5.33 errors (*SD* = 1.95) in the WM task in the simulator condition and on average 8.25 (*SD* = 2.42) errors in the real flight condition, all occurring during the high load trials (see Figure 9). As no error was committed in the low WM load condition, we performed a statistical analysis to compare the effect of the flight conditions on WM performance in the high load conditions. An unpaired t-test revealed that the real flight condition led to significantly higher number of errors on the WM task in the high load condition (*p* < 0.001, *Cohen*′*sd* = 1.34). The MANOVA over the mean Δ[*HbO*_2_] data disclosed a significant WM load × Flight condition × ROI interaction [*F*_(3, 66)_ = 3.36; *p* = 0.039; see Figure 10]. *Post- hoc* analyses revealed that high load trials performed in real flight condition led to higher Δ[*HbO*_2_] in ROI #2 than their counterparts performed in simulator (*p* = 0.0001). The MANOVA over the mean Δ[*hHb*] data did not disclose any significant WM load × Flight condition × ROI interaction [*F*_(3, 66)_ = 0.69; *p* = 0.56].

### 3.2. Single trial SVM-based WM load estimation results

#### 3.2.1. Simulator

During the testing phase, a mean of 76.66% (*SD*:16.14%) of the trials were accurately classified (discriminated into on-line low WM load trials and high WM load trials). We obtained a 85.60% mean precision (*SD*:19.36%) and a 73.33% mean recall (*SD* = 24.62%). Individual classifiers' accuracies are shown in Table 1.

**Table 1:** Simulator experiment: machine learning results.

**Subject**	**Accuracy training**	**Accuracy testing**	**Precision**	**Recall**	**f1 score**
01	90.00	100.00	100.00	100.00	100.00
02	100.00	60.00	66.67	40.00	50.00
03	100.00	70.00	100.00	40.00	57.14
04	100.00	100.00	100.00	100.00	100.00
05	100.00	70.00	75.00	60.00	66.67
06	100.00	80.00	100.00	60.00	75.00
07	100.00	90.00	100.00	80.00	88.89
08	100.00	80.00	80.00	80.00	80.00
09	100.00	90.00	100.00	80.00	88.89
10	80.00	70.00	100.00	40.00	57.14
11	70.00	50.00	50.00	100.00	66.67
12	90.00	60.00	55.56	100.00	71.43

#### 3.2.2. Real flight

During the testing phase, a mean of 78.33% (*SD*:11.93%) of the trials were accurately classified (discriminated into on-line low WM load trials and high WM load trials). We obtained a 84.14% mean precision (*SD*:18.56%) and a 76.67% mean recall (*SD* = 22.29%). Individual classifiers' accuracies are shown in Table 2.

**Table 2:** Real flight experiment: machine learning results.

**Subject**	**Accuracy training**	**Accuracy testing**	**Precision**	**Recall**	**f1 score**
01	70.0	80.0	71.4	100	83.3
02	100	90.0	100	80.0	88.9
03	100	70.0	62.5	100	76.9
04	100	80.0	100	60.0	75.0
05	100	90.0	100	80.0	88.9
06	100	90.0	100	80.0	88.9
07	90.0	50.0	50.0	40.0	44.4
08	100	70.0	62.5	100	76.9
09	100	70.0	100	40.0	57.1
10	100	80.0	100	60.0	75.0
11	80.0	80.0	80.0	80.0	80.0
12	100	90.0	83.3	100	90.9

#### 3.2.3. Real flight vs. flight simulator: statistical analysis

A t-test disclosed no statistical differences of the classification accuracy in the two experimental conditions (*p* = 0.67, *Cohen*′*s* d = 0.17).

## 4. Discussion

The motivation of this study was to develop on-line tools to monitor pilots' cognitive performance under realistic settings. We followed a two-step methodological approach as we first implemented and tested an inference system in a flight simulator and then in a real aircraft. We designed a task known to elicit WM (Durantin et al., 2015; Gateau et al., 2015) as this executive function is particularly engaged when operating aircraft (Causse et al., 2011a,b).

### 4.1. Summary of findings

The behavioral results confirmed that these two levels of WM load were well contrasted, as the participants exhibited lower performance during the higher difficulty level. This result is in line with Taylor et al. study (Taylor et al., 2005; Durantin et al., 2015) and previous experiments (Gateau et al., 2015) showing that pilots' WM performance decline when four different ATC instructions have to be read back. Moreover, this drop in performance was most significant for the participants under actual flight conditions. Consistent with this finding, the real flight condition yielded to higher PFC activation than the simulated one only when the pilots had to execute the difficult WM load task. Taken together, these findings suggest that the mental demand was higher when operating the actual aircraft as the participants had not only to perform the WM task but also to monitor the flight path, the aircraft status and the airspace in a much more careful fashion than in the simulated condition.

Whereas this multitasking aspect of the real flight was not detrimental from a behavioral and neurophysiological point of view when performing the low WM stimuli it became critical when engaged in the high WM one. One could suspect prioritization issue leading the pilots to focus more on flying the aircraft thus leaving few resources available to face the demand of the high WM stimuli. This could be one explanation for the higher levels of activation observed in fNIRS measurements that reflect the higher load of concurrent cognitive tasks induced by the real flying task compared to the simulated. Unfortunately, our aircraft was not equipped with a flight data recorder preventing us from analyzing the flight performance and investigating these prioritization and multi-tasking issues. Despite this limit, our study is consistent with Dahlstrom and Nahlinder (2009) who found evidence of higher cardiac activity when flying under realistic settings than in flight simulator. These results raise the question of the ecological validity of simulators. Their use is of undeniable interest (e.g., understanding cognitive performance, training pilots, assessing cockpit design) and they present several advantages in terms of economical costs and reproducibility issues. However, our findings and others (Philip et al., 2005; Dahlstrom and Nahlinder, 2009) suggest that the simulators may need to be calibrated against real flying conditions to be more engaging.

Several field studies have demonstrated the potential of fNIRS to measure cortical activity while walking outdoors (McKendrick et al., 2016), facing prolonged stay at high altitude (Davranche et al., 2016), riding bikes (Piper et al., 2014), motorcycles (Kawashima et al., 2014), and flying helicopters (Kikukawa et al., 2008). Our study was conducted in accordance with the recent neuroergonomics approach to measure brain activity out of the laboratory. Indeed, beyond the offline analyses, we used machine learning techniques to perform single trial discrimination of the low WM load versus high WM load trials. The results of the classification process were available and displayed in a terminal to the experimenter after each: as soon as data of the trial were available, SVM discrimination process never required more than 10 ms to provide its result. The mean accuracy to classify low vs. high WM trials in the two experimental conditions exceeded the threshold of 70%, defined as a sufficient rate for pBCI (Kubler et al., 2006; Tai and Chau, 2009). These results compare well to the rare on-line studies such as the ones conducted by Naseer et al. (2014) (14 participants: 82.14% accuracy), Girouard et al. (2013) (9 participants: 83.5% accuracy), and (Schudlo and Chau, 2014) (10 participants: 77.4% accuracy). However, these and other (Kanoh et al., 2009; Hu et al., 2012; Power et al., 2012; Robinson et al., 2016) fNIRS-based BCI were not implemented under realistic settings and describe experiments in controlled lab settings.

### 4.2. Limitations and avenues for future research

Despite the promising results presented in this paper for development of fNIRS based pBCI in ecologically valid environment, one could argue that the translation of the fNIRS-based pBCI in real cockpit to day-to-day flight operations might not be applicable. First, the addition of machine learning and this on-line classifier approach to standard procedures of aviation still remains a challenge as the reliability of the classifier does not meet aviation certification criteria (10^−3^ allowable failure probability). One approach to overcome this reliability problem would be to integrate complementary measurements such as EEG that could significantly enhance classification performance when combined with fNIRS as suggested by Khan et al. (2014).

Also, the accuracy score per subject must be interpreted with caution. In a two classes and five testing trials per class to fit with experimental constraints, classification performance should be higher than 75% to be statistically significant (*p* < 0.05) (Müller-Putz et al., 2008; Combrisson and Jerbi, 2015). Considering both groups in this study, 17 of 24 subjects were already above this threshold with our online classifier. Further improvements with machine learning methodologies would be needed to improve and optimize the classifier performance.

Secondly, availability of the information about WM level estimation is a key preoccupation. One criteria to evaluate on-line inference system is related to the delay of single trial classification. In our study, the diagnosis of the WM lasted less than 1.01 s after each pilot's response window. It could allow, for instance, to automatically give a feedback to ATC that the pilot is currently facing a high workload situation and may have misinterpreted the last communication. This timing was comparable with results from other on-line fNIRS-based BCI latency (for a review of on-line fNIRS-based BCI latency, please see Strait et al., 2014). However, solutions have to be explored to speed up response detection on fNIRS signal that can drastically reduce latency in detecting change in a mental state (Cui et al., 2010; Hong and Naseer, 2016). Thirdly, our study was limited to monitor WM load in a binary and discrete fashion. Further studies have to be conducted to continuously discriminate a gradient of WM levels from underload to overload (Unni et al., 2017). Eventually, lingering issues remain regarding the effect of accelerations and headband motion on fNIRS signal (Mackey et al., 2013). In other scenarios accelerometer data with special processing could be used to eliminate any systemic effect of blood pooling.

Also, one should consider that fNIRS based pBCI could be first used for civilian application as highly automated modern aircraft prevents pilots from exceeding 1g maneuvers for passenger comfort and to avoid going against the *flight envelope protection*. Despite these limits, one can propose a progressive framework for the introduction of fNIRS in aviation. A first step is to consider the use of fNIRS based BCI to improve training via neurofeedback (Pope et al., 2014) and to tailor the flight sessions to the trainee (Chad et al., 2018). A second step is to use such inference system to monitor pilot's brain activity during each operational flight for *quantified self* purpose. These daily measures can be used to assess pilot's cognitive workload state and mental fatigue thus providing airlines with analyses tools for crew rostering. A third step is to stream the fNIRS data to the flight data recorder for accident analyses. These logged neurophysiological data would provide additional insights on the crew's cognition during these critical events and help accident investigators. A last step, when the reliability of the fNIRS-based inference system will meet the standard, would be to adapt the flight deck depending on the crew's changing WM load level. As previously demonstrated, stochastic decisional systems could be implemented to infer that human operators are engaged in demanding WM task and dynamically adapt interactions to prevent them from distraction (Gateau et al., 2016). The objective is to improve task allocation to enable better task switching, interruption management, and multi-tasking (Kohlmorgen et al., 2007; Solovey et al., 2011). Eventually, one should consider that such fNIRS based system could be applied to variety of contexts whereby human operators interact with complex and critical systems (e.g., nuclear powerplant, train).

In summary, this study is the first report of the use of an online fNIRS based pBCI both in simulation (in silico) and in aircraft during flight (over the clouds) to measure pilot's WM. The implementation of this pBCI led to address several technical constraints, adapting and testing for instance a new wireless fNIRS that can be used by pilots and that has been approved for use during real flight. It also led to identify solutions to address potential sources of noise in signals such as the sunlight infrared shielding using aluminum based cover. Moreover, it provides important albeit preliminary information about fNIRS measures of the PFC hemodynamic response and its relationship to working memory workload, and in both simulation and actual flight environment. Level of immersion or realistic aspect of flight environment does appear to influence the performance as well as hemodynamic response in the anterior prefrontal cortex, at least for the air traffic control related working memory task. The measurements in simulator had larger fNIRS sensor coverage and future studies may compare simulation vs. actual flight or level of realistic aspect of environment with larger cortical coverage within the actual flight environment, for a more granular detailed comparison. Since fNIRS technology allows the development of mobile, non-intrusive and miniaturized devices, it has the potential to be deployed in future operational environments to monitor the pilot, adapt the complex system interface, and/or to assess the training of operators.

## Author contributions

Study conception and design: FD, TG, and HA. Data acquisition : TG and FD. Data analysis : TG, FD, and HA. Data interpretation and writing FD, HA, and TG.

### Conflict of interest statement

fNIR Devices, LLC manufactures the optical brain imaging instrument and licensed IP and know-how from Science Health Systems, Drexel University. HA was involved in the technology development and thus offered a minor share in the startup firm fNIR Devices, LLC. The other authors declare that the research was conducted in the absence of any commercial or financial relationships that could be construed as a potential conflict of interest.

## References

[B1] Anderson M. J. (2001). A new method for non-parametric multivariate analysis of variance. Austral Ecol. 26, 32–46. 10.1111/j.1442-9993.2001.01070.pp.x

[B2] Appel G. (2005). Technical Analysis: Power Tools for Active Investors. FT Press.

[B3] Aricò P. Borghini G. Di Flumeri G. Colosimo A. Pozzi S. Babiloni F. (2016). A passive brain–computer interface application for the mental workload assessment on professional air traffic controllers during realistic air traffic control tasks. Prog. Brain Res. 228, 295–328. 10.1016/bs.pbr.2016.04.021 27590973

[B4] Ayaz H. Onaral B. (2005). Analytical Software and Stimulus-Presentation Platform to Utilize, Visualize and Analyze Near-Infrared Spectroscopy Measures. Master's thesis, Master's Degree Thesis, Drexel University.

[B5] Ayaz H. Onaral B. Izzetoglu K. Shewokis P. A. McKendrick R. Parasuraman R. (2013). Continuous monitoring of brain dynamics with functional near infrared spectroscopy as a tool for neuroergonomic research: empirical examples and a technological development. Front. Hum. Neurosci. 7:871. 10.3389/fnhum.2013.00871 24385959 PMC3866520

[B6] Ayaz H. Shewokis P. A. Bunce S. Izzetoglu K. Willems B. Onaral B. (2012). Optical brain monitoring for operator training and mental workload assessment. Neuroimage 59, 36–47. 10.1016/j.neuroimage.2011.06.023 21722738

[B7] Ayaz H. Shewokis P. A. Curtin A. Izzetoglu M. Izzetoglu K. Onaral B. (2011). Using mazesuite and functional near infrared spectroscopy to study learning in spatial navigation. J. Vis. Exp. e3443. 10.3791/3443 22005455 PMC3227178

[B8] Baddeley A. (1992). Working memory. Science 255, 556–559. 1736359 10.1126/science.1736359

[B9] Batula A. M. Kim Y. E. Ayaz H. (2017). Virtual and actual humanoid robot control with four-class motor-imagery-based optical brain-computer interface. BioMed. Res. Int. 2017:1463512. 10.1155/2017/1463512 28804712 PMC5539938

[B10] Billings C. E. Cheaney E. S. (1981). Information Transfer Problems in the Aviation System. NASA Technical Report 1875, 89–90.

[B11] Blankertz B. Tangermann M. Vidaurre C. Fazli S. Sannelli C. Haufe S.. (2010). The berlin brain–computer interface: non-medical uses of bci technology. Front. Neurosci. 4:198. 10.3389/fnins.2010.00198 21165175 PMC3002462

[B12] Borghini G. Aricò P. Di Flumeri G. Cartocci G. Colosimo A. Bonelli S.. (2017). EEG-based cognitive control behaviour assessment: an ecological study with professional air traffic controllers. Sci. Rep. 7:547. 10.1038/s41598-017-00633-7 28373684 PMC5428823

[B13] Brouwer A.-M. Hogervorst M. A. Van Erp J. B. Heffelaar T. Zimmerman P. H. Oostenveld R. (2012). Estimating workload using eeg spectral power and erps in the n-back task. J. Neural Eng. 9:045008. 10.1088/1741-2560/9/4/045008 22832068

[B14] Brouwer A.-M. Van Erp J. Heylen D. Jensen O. Poel M. (2013). Effortless passive bcis for healthy users, in Universal Access in Human-Computer Interaction. Design Methods, Tools, and Interaction Techniques for eInclusion, Vol. 8009 (Berlin; Heidelberg; Vancouver: Springer), 615–622.

[B15] Çakır M. P. Vural M. Koç S. Ö. Toktaş A. (2016). Real-time monitoring of cognitive workload of airline pilots in a flight simulator with fnir optical brain imaging technology, in International Conference on Augmented Cognition (Springer), 147–158.

[B16] Callan D. E. Durantin G. Terzibas C. (2015). Classification of single-trial auditory events using dry-wireless eeg during real and motion simulated flight. Front. Syst. Neurosci. 9:11. 10.3389/fnsys.2015.00011 25741249 PMC4330719

[B17] Callan D. E. Terzibas C. Cassel D. B. Sato M.-A. Parasuraman R. (2016). The brain is faster than the hand in split-second intentions to respond to an impending hazard: a simulation of neuroadaptive automation to speed recovery to perturbation in flight attitude. Front. Hum. Neurosci. 10:187. 10.3389/fnhum.2016.00187 27199710 PMC4846799

[B18] Causse M. Dehais F. Arexis M. Pastor J. (2011a). Cognitive aging and flight performances in general aviation pilots. Aging Neuropsychol. Cogn. 18, 544–561. 10.1080/13825585.2011.586018 21819276

[B19] Causse M. Dehais F. Pastor J. (2011b). Executive functions and pilot characteristics predict flight simulator performance in general aviation pilots. Int. J. Aviat. Psychol. 21, 217–234. 10.1080/10508414.2011.582441

[B20] Causse M. Dehais F. Péran P. Sabatini U. Pastor J. (2013). The effects of emotion on pilot decision-making: a neuroergonomic approach to aviation safety. Transport. Res. Part C Emerg. Technol. 33, 272–281. 10.1016/j.trc.2012.04.005

[B21] Chad S. Dehais F. Roy N. R. Harrivel A. Last M. C. Kennedy K. (2018). Biocybernetic Adaptation Strategies: Machine Awareness of Human Engagement for Improved Operational Performance, in HCI Conference (Las Vegas).

[B22] Combrisson E. Jerbi K. (2015). Exceeding chance level by chance: the caveat of theoretical chance levels in brain signal classification and statistical assessment of decoding accuracy. J. Neurosci. Methods 250, 126–136. 10.1016/j.jneumeth.2015.01.010 25596422

[B23] Cortes C. Vapnik V. (1995). Support-vector networks. Mach. Learn. 20, 273–297.

[B24] Cui X. Bray S. Reiss A. L. (2010). Speeded near infrared spectroscopy (NIRS) response detection. PLoS ONE 5:e15474. 10.1371/journal.pone.0015474 21085607 PMC2978722

[B25] Cutrell E. Tan D. (2008). BCI for passive input in HCI. in Proc. CHI 8, 1–3.

[B26] Dahlstrom N. Nahlinder S. (2009). Mental workload in aircraft and simulator during basic civil aviation training. Int. J. Aviat. Psychol. 19, 309–325. 10.1080/10508410903187547

[B27] Davranche K. Casini L. Arnal P. J. Rupp T. Perrey S. Verges S. (2016). Cognitive functions and cerebral oxygenation changes during acute and prolonged hypoxic exposure. Physiol. Behav. 164, 189–197. 10.1016/j.physbeh.2016.06.001 27262217

[B28] Dehais F. Behrend J. Peysakhovich V. Causse M. Wickens C. D. (2017). Pilot flying and pilot monitoring aircraft state awareness during go-around execution in aviation: a behavioral and eye tracking study. Int. J. Aerospace Psychol. 27, 15–28. 10.1080/10508414.2017.1366269

[B29] Delpy D. T. Cope M. Van der Zee P. Arridge S. Wray S. Wyatt J. (1988). Estimation of optical pathlength through tissue from direct time of flight measurement. Phys. Med. Biol. 33:1433. 3237772 10.1088/0031-9155/33/12/008

[B30] Dijksterhuis C. de Waard D. Brookhuis K. Mulder B. de Jong R. (2013). Classifying visuomotor workload in a driving simulator using subject specific spatial brain patterns. Front. Neurosci. 7:149. 10.3389/fnins.2013.00149 23970851 PMC3748749

[B31] Durantin G. Gagnon J.-F. Tremblay S. Dehais F. (2014a). Using near infrared spectroscopy and heart rate variability to detect mental overload. Behav. Brain Res. 259, 16–23. 10.1016/j.bbr.2013.10.042 24184083

[B32] Durantin G. Scannella S. Gateau T. Delorme A. Dehais F. (2014b). Moving average convergence divergence filter preprocessing for real-time event-related peak activity onset detection: Application to fnirs signals, in Engineering in Medicine and Biology Society (EMBC), 2014 36th Annual International Conference of the IEEE (Chicago, IL: IEEE), 2107–2110. 10.1109/EMBC.2014.6944032 25570400

[B33] Durantin G. Scannella S. Gateau T. Delorme A. Dehais F. (2015). Processing functional near infrared spectroscopy signal with a kalman filter to assess working memory during simulated flight. Front. Hum. Neurosci. 9:707. 10.3389/fnhum.2015.00707 26834607 PMC4719469

[B34] Frey J. Hachet M. Lotte F. (2017). EEG-based neuroergonomics for 3D user interfaces: opportunities and challenges. Le travail humain. 80, 73–92.

[B35] Gagnon J.-F. Durantin G. Vachon F. Causse M. Tremblay S. Dehais F. (2012). Anticipating human error before it happens: towards a psychophysiological model for online prediction of mental workload, in Proceedings of the Human Factors and Ergonomics Society Chapter Europe.

[B36] Gateau T. Durantin G. Lancelot F. Scannella S. Dehais F. (2015). Real-time state estimation in a flight simulator using fNIRS. PLoS ONE 10:e0121279. 10.1371/journal.pone.0121279 25816347 PMC4376943

[B37] Gateau T. P. Carvalho Chanel C. Le M.-H. Dehais F. (2016). Considering human's non-deterministic behavior and his availability state when designing a collaborative human-robots system, in 2016 IEEE/RSJ International Conference on Intelligent Robots and Systems (Daejeon: IEEE).

[B38] George L. Lécuyer A. (2010). An overview of research on” passive” brain-computer interfaces for implicit human-computer interaction, in International Conference on Applied Bionics and Biomechanics ICABB 2010-Workshop W1” Brain-Computer Interfacing and Virtual Reality (Venice).

[B39] Girouard A. Solovey E. T. Jacob R. J. (2013). Designing a passive brain computer interface using real time classification of functional near-infrared spectroscopy. Inte. J. Auton. Adapt. Commun. Syst. 6, 26–44. 10.1504/IJAACS.2013.050689

[B40] Gramann K. Fairclough S. H. Zander T. O. Ayaz H. (2017). Editorial: trends in neuroergonomics. Front. Hum. Neurosci. 11:165. 10.3389/fnhum.2017.00165 28424601 PMC5380744

[B41] Herff C. Heger D. Fortmann O. Hennrich J. Putze F. Schultz T. (2014). Mental workload during n-back task?quantified in the prefrontal cortex using fNIRS. Front. Hum. Neurosci. 7:935. 10.3389/fnhum.2013.00935. 24474913 PMC3893598

[B42] Hirshfield L. M. Gulotta R. Hirshfield S. Hincks S. Russell M. Ward R. (2011). This is your brain on interfaces: enhancing usability testing with functional near-infrared spectroscopy, in Proceedings of the SIGCHI Conference on Human Factors in Computing Systems (ACM), 373–382.

[B43] Hong K.-S. Naseer N. (2016). Reduction of delay in detecting initial dips from functional near-infrared spectroscopy signals using vector-based phase analysis. Int. J. Neural syst. 26:1650012. 10.1142/S012906571650012X 26971785

[B44] Hu X.-S. Hong K.-S. Ge S. S. (2012). fNIRS-based online deception decoding. J. Neural Eng. 9:026012. 10.1088/1741-2560/9/2/026012 22337819

[B45] Huppert T. J. Diamond S. G. Franceschini M. A. Boas D. A. (2009). Homer: a review of time-series analysis methods for near-infrared spectroscopy of the brain. Appl. Opt. 48, D280–D298. 10.1364/AO.48.00D280 19340120 PMC2761652

[B46] Izzetoglu K. Bunce S. Onaral B. Pourrezaei K. Chance B. (2004). Functional optical brain imaging using near-infrared during cognitive tasks. Int. J. Hum. Comput. Interact. 17, 211–227. 10.1207/s15327590ijhc1702_6

[B47] Jasdzewski G. Strangman G. Wagner J. Kwong K. Poldrack R. Boas D. (2003). Differences in the hemodynamic response to event-related motor and visual paradigms as measured by near-infrared spectroscopy. Neuroimage 20, 479–488. 10.1016/S1053-8119(03)00311-2 14527608

[B48] Kanoh S. Murayama Y.-M. Miyamoto K.-I. Yoshinobu T. Kawashima R. (2009). A NIRS-based brain-computer interface system during motor imagery: System development and online feedback training, in 2009 Annual International Conference of the IEEE Engineering in Medicine and Biology Society (Minneapolis: IEEE), 594–597. 10.1109/IEMBS.2009.5333710 19964231

[B49] Kato T. Kamei A. Takashima S. Ozaki T. (1993). Human visual cortical function during photic stimulation monitoring by means of near-infrared spectroscopy. J. Cereb. Blood Flow Metab. 13, 516–520. 8478409 10.1038/jcbfm.1993.66

[B50] Kawashima R. Matsumoto T. Tanimoto Y. (2014). Cortical activity while riding motorcycles measured with a wearable near infrared topography system. Int. J. Automot. Eng. 5, 77–83. 10.20485/jsaeijae.5.2_77

[B51] Khan M. J. Hong M. J. Hong K.-S. (2014). Decoding of four movement directions using hybrid NIRS-EEG brain-computer interface. Front. Hum. Neurosci. 8:244. 10.3389/fnhum.2014.00244 24808844 PMC4009438

[B52] Kikukawa A. Kobayashi A. Miyamoto Y. (2008). Monitoring of pre-frontal oxygen status in helicopter pilots using near-infrared spectrophotometers. Dyn. Med. 7:10. 10.1186/1476-5918-7-10 18616829 PMC2503955

[B53] Kohlmorgen J. Dornhege G. Braun M. Blankertz B. Müller K.-R. Curio G. (2007). Improving human performance in a real operating environment through real-time mental workload detection, in Toward Brain-Computer Interfacing (Cambridge, MA: MIT press), 409–422.

[B54] Kubler A. Mushahwar V. Hochberg L. R. Donoghue J. P. (2006). BCI meeting 2005-workshop on clinical issues and applications. IEEE Trans. Neural Syst. Rehabil. Eng. 14, 131–134. 10.1109/TNSRE.2006.875585 16792277

[B55] León-Domínguez U. Martín-Rodríguez J. F. León-Carrión J. (2015). Executive n-back tasks for the neuropsychological assessment of working memory. Behav. Brain Res. 292, 167–173. 10.1016/j.bbr.2015.06.002 26068585

[B56] Li C. Gong H. Zeng S. Luo Q. (2005). Verbal Working Memory Load Affects Prefrontal Cortices Activation: Evidence from a Functional Nirs Study in Humans (San Diego, CA: In SPIE Conference on Optics and Photonics), 33–40.

[B57] Luo Q. Zeng S. Chance B. Nioka S. (2002). Monitoring of brain activity with near-infrared spectroscopy, in Handbook of Optical Biomedical Diagnostics (Bellingham, WA: SPIE Press), 455–486. 10.1117/3.2219603.ch8

[B58] Mackey J. R. Harrivel A. R. Adamovsky G. Lewandowski B. E. Gotti D. J. Tin P. (2013). Effects of varying gravity levels on fnirs headgear performance and signal recovery, in The American Institute of Aeronautics and Astronautics (Boston, MA).

[B59] McKendrick R. Ayaz H. Olmstead R. Parasuraman R. (2014). Enhancing dual-task performance with verbal and spatial working memory training: continuous monitoring of cerebral hemodynamics with NIRS. Neuroimage 85, 1014–1026. 10.1016/j.neuroimage.2013.05.103 23727530

[B60] McKendrick R. Parasuraman R. Ayaz H. (2015). Wearable functional near infrared spectroscopy (fnirs) and transcranial direct current stimulation (tdcs): expanding vistas for neurocognitive augmentation. Front. Syst. Neurosci. 9:27. 10.3389/fnsys.2015.00027 25805976 PMC4353303

[B61] McKendrick R. Parasuraman R. Murtza R. Formwalt A. Baccus W. Paczynski M.. (2016). Into the wild: neuroergonomic differentiation of hand-held and augmented reality wearable displays during outdoor navigation with functional near infrared spectroscopy. Front. Hum. Neurosci. 10:216. 10.3389/fnhum.2016.00216 27242480 PMC4870997

[B62] Miller G. A. (1994). The magical number seven, plus or minus two: Some limits on our capacity for processing information. Psychol. Rev. 101:343. 8022966 10.1037/0033-295x.101.2.343

[B63] Morrow D. Lee A. Rodvold M. (1993). Analysis of problems in routine controller-pilot communication. Int. J. Aviat. Psychol. 3, 285–302.

[B64] Mühl C. Jeunet C. Lotte F. (2014). EEG-based workload estimation across affective contexts. Front. Neurosci. 8:114. 10.3389/fnins.2014.00114 24971046 PMC4054975

[B65] Müller-Putz G. Scherer R. Brunner C. Leeb R. Pfurtscheller G. (2008). Better than random: a closer look on bci results. Int. J. Bioelectromagnet. 10, 52–55.

[B66] Naseer N. Hong K.-S. (2015). fNIRS-based brain-computer interfaces: a review. Front. Hum. Neurosci. 9:3. 10.3389/fnhum.2015.00003 25674060 PMC4309034

[B67] Naseer N. Hong M. J. Hong K.-S. (2014). Online binary decision decoding using functional near-infrared spectroscopy for the development of brain–computer interface. Exp. Brain Res. 232, 555–564. 10.1007/s00221-013-3764-1 24258529

[B68] Parasuraman R. Rizzo M. (2008). Neuroergonomics: The Brain at Work. New York, NY: Oxford University Press.

[B69] Parasuraman R. Wilson G. F. (2008). Putting the brain to work: Neuroergonomics past, present, and future. Hum. Factors 50, 468–474. 10.1518/001872008X288349 18689055

[B70] Pedregosa F. Varoquaux G. Gramfort A. Michel V. Thirion B. Grisel O. (2011). Scikit-learn: machine learning in Python. J. Mach. Learn. Res. 12, 2825–2830. 10.1016/j.patcog.2011.04.006

[B71] Philip P. Sagaspe P. Taillard J. Valtat C. Moore N. Akerstedt T.. (2005). Fatigue, sleepiness, and performance in simulated versus real driving conditions. Sleep 28:1511. 10.1093/sleep/28.12.1511 16408409

[B72] Piper S. K. Krueger A. Koch S. P. Mehnert J. Habermehl C. Steinbrink J.. (2014). A wearable multi-channel fNIRS system for brain imaging in freely moving subjects. Neuroimage 85, 64–71. 10.1016/j.neuroimage.2013.06.062 23810973 PMC3859838

[B73] Pope A. T. Stephens C. L. Gilleade K. (2014). Biocybernetic adaptation as biofeedback training method, in Advances in Physiological Computing, eds Fairclough S. Gilleade K. (London: Springer), 91–115.

[B74] Power S. D. Kushki A. Chau T. (2012). Intersession consistency of single-trial classification of the prefrontal response to mental arithmetic and the no-control state by NIRS. PLoS ONE 7:e37791. 10.1371/journal.pone.0037791 22844390 PMC3402505

[B75] R Core Team (2013). R: A Language and Environment for Statistical Computing. Vienna: R Foundation for Statistical Computing.

[B76] Reynal M. Colineaux Y. Vernay A. Dehais F. (2016). Pilot flying vs. pilot monitoring during the approach phase: an eye–tracking study, in HCI-Aero 2016, International Conference on Human-Computer Interaction in Aerospace (Paris).

[B77] Risser M. R. Scerbo M. W. Baldwin C. L. McNamara D. S. (2006). Interference timing and acknowledgement response with voice and datalink atc commands, in Proceedings of the Human Factors and Ergonomics Society Annual Meeting, Vol. 50 (San Francisco, CA: Sage Publications), 11–15.

[B78] Robinson N. Zaidi A. D. Rana M. Prasad V. A. Guan C. Birbaumer N.. (2016). Real-time subject-independent pattern classification of overt and covert movements from fNIRS signals. PLoS ONE 11:e0159959. 10.1371/journal.pone.0159959 27467528 PMC4965045

[B79] Rome F. Adam G. Condette J. Causse M. Dehais F. (2012). Go-around manoeuver: a simulation study, in Proceedings of the European Association For Aviation Psychology Conference (Lisboa: SAGE Publications).

[B80] Roy R. N. Bonnet S. Charbonnier S. Campagne A. (2013). Mental fatigue and working memory load estimation: interaction and implications for eeg-based passive bci, in Engineering in Medicine and Biology Society (EMBC), 2013 35th Annual International Conference of the IEEE (Osaka: IEEE), 6607–6610. 10.1109/EMBC.2013.6611070 24111257

[B81] Roy R. N. Verdiere K. Scannella S. Dehais F. (2017). Passive BCI tools for mental state estimation in aerospace applications, in The First Biannual Neuroadaptive Technology Conference (Berlin), 79.

[B82] Sato H. Fuchino Y. Kiguchi M. Katura T. Maki A. Yoro T.. (2005). Intersubject variability of near-infrared spectroscopy signals during sensorimotor cortex activation. J. Biomed. Opt. 10:044001. 10.1117/1.1960907 16178635

[B83] Scerbo M. W. Risser M. R. Baldwin C. L. (2003). Implementing speech and simulated data link commands: the role of task interference and message length, in Proceedings of the Human Factors and Ergonomics Society Annual Meeting, Vol. 47 (Denver: SAGE Publications), 95–99.

[B84] Schreppel T. Egetemeir J. Schecklmann M. Plichta M. M. Pauli P. Ellgring H. Fallgatter A. J. Herrmann M. J. (2008). Activation of the prefrontal cortex in working memory and interference resolution processes assessed with near-infrared spectroscopy. Neuropsychobiology, 57, 188–193. 18654088 10.1159/000147473

[B85] Schudlo L. C. Chau T. (2014). Dynamic topographical pattern classification of multichannel prefrontal NIRS signals: II. online differentiation of mental arithmetic and rest. J. Neural Eng. 11:016003. 10.1088/1741-2560/11/1/016003 24311057

[B86] Schudlo L. C. Chau T. (2015). Towards a ternary NIRS-bci: single-trial classification of verbal fluency task, stroop task and unconstrained rest. J. Neural Eng. 12:066008. 10.1088/1741-2560/12/6/066008 26447770

[B87] Solovey E. T. Lalooses F. Chauncey K. Weaver D. Parasi M. Scheutz M. (2011). Sensing cognitive multitasking for a brain-based adaptive user interface, in Proceedings of the SIGCHI Conference on Human Factors in Computing Systems (Vancouver, BC: ACM), 383–392.

[B88] Strait M. Canning C. Scheutz M. (2014). What we can and cannot do with near infrared spectroscopy. Front. Neurosci. 8:117. 10.3389/fnins.2014.00117 24904261 PMC4033094

[B89] Tai K. Chau T. (2009). Single-trial classification of nirs signals during emotional induction tasks: towards a corporeal machine interface. J. Neuroeng. Rehabil. 6:39. 10.1186/1743-0003-6-39 19900285 PMC2779792

[B90] Taylor J. L. O'Hara R. Mumenthaler M. S. Rosen A. C. Yesavage J. A. (2005). Cognitive ability, expertise, and age differences in following air-traffic control instructions. Psychol. Aging 20, 117–133. 10.1037/0882-7974.20.1.117 15769218

[B91] Taylor J. L. Yesavage J. A. Morrow D. G. Dolhert N. Brooks J. O. Poon L. W. (1994). The effects of information load and speech rate on younger and older aircraft pilots' ability to execute simulated air-traffic controller instructions. J. Gerontol. 49, 191–200. 8056944 10.1093/geronj/49.5.p191

[B92] Unni A. Ihme K. Jipp M. Rieger J. W. (2017). Assessing the driver?s current level of working memory load with high density functional near-infrared spectroscopy: a realistic driving simulator study. Front. Hum. Neurosci. 11:167. 10.3389/fnhum.2017.00167 28424602 PMC5380755

[B93] Utsugi K. Obata A. Sato H. Katsura T. Sagara K. Maki A.. (2007). Development of an optical brain-machine interface, in Engineering in Medicine and Biology Society, 2007. EMBS 2007. 29th Annual International Conference of the IEEE (IEEE), 5338–5341. 10.1109/IEMBS.2007.4353547 18003213

[B94] Van Erp J. Brouwer A. Zander T. (2015). Editorial: Using neurophysiological signals that reflect cognitive or affective state. Front. Neurosci. 9:193. 10.3389/fnins.2015.00193 26074763 PMC4448037

[B95] Vecchiato G. Borghini G. Aricò P. Graziani I. Maglione A. G. Cherubino P.. (2016). Investigation of the effect of eeg-bci on the simultaneous execution of flight simulation and attentional tasks. Med. Biol. Eng. Comput. 54, 1503–1513. 10.1007/s11517-015-1420-6 26645694

[B96] Verdière K. J. Roy R. N. Dehais F. (2018). Detecting pilot's engagement using fnirs connectivity features in an automated vs manual landing scenario. Front. Hum. Neurosci. 12:6. 10.3389/fnhum.2018.00006 29422841 PMC5788966

[B97] Villringer A. Obrig H. (2002). Near infrared spectroscopy and imaging, in Brain Mapping: The Methods, 2nd edn. (Sendai: Elsevier), 141–158.

[B98] Zander T. O. Kothe C. (2011). Towards passive brain–computer interfaces: applying brain–computer interface technology to human–machine systems in general. J. Neural Eng. 8:025005. 10.1088/1741-2560/8/2/025005 21436512

[B99] Zander T. O. Shetty K. Lorenz R. Leff D. R. Krol L. R. Darzi A. W. Gramann K. Yang G.-Z. (2017). Automated task load detection with electroencephalography: Towards passive brain–computer interfacing in robotic surgery. J. Med. Robot. Res. 2:1750003 10.1142/S2424905X17500039

